# Enhanced Design of Gold Catalysts for Bioorthogonal Polyzymes

**DOI:** 10.3390/ma15186487

**Published:** 2022-09-19

**Authors:** Cristina-Maria Hirschbiegel, Stefano Fedeli, Xianzhi Zhang, Rui Huang, Jungmi Park, Yisheng Xu, Vincent M. Rotello

**Affiliations:** 1 Department of Chemistry, University of Massachusetts Amherst, 710 North Pleasant Street, Amherst, MA 01003, USA; 2 State Key Laboratory of Chemical Engineering, East China University of Science and Technology, Shanghai 200237, China

**Keywords:** bioorthogonal catalysis, gold catalysts, polymeric nanoparticles, catalyst design

## Abstract

Bioorthogonal chemistry introduces nonbiogenic reactions that can be performed in biological systems, allowing for the localized release of therapeutic agents. Bioorthogonal catalysts can amplify uncaging reactions for the in situ generation of therapeutics. Embedding these catalysts into a polymeric nanoscaffold can protect and modulate the catalytic activity, improving the performance of the resulting bioorthogonal “polyzymes”. Catalysts based on nontoxic metals such as gold(I) are particularly attractive for therapeutic applications. Herein, we optimized the structural components of a metal catalyst to develop an efficient gold(I)-based polyzyme. Tailoring the ligand structure of gold phosphine-based complexes, we improved the affinity between the metal complex and polymer scaffold, resulting in enhanced encapsulation efficiency and catalytic rate of the polyzyme. Our findings show the dependence of the overall polyzyme properties on the structural properties of the encapsulated metal complex.

## 1. Introduction

Bioorthogonal chemistry enables the performance of chemical reactions that are not native to a cell or living environment [1]. Bioorthogonal catalysis provides the in situ generation of drug molecules from their caged and nontoxic prodrug counterparts [2]. This strategy is particularly promising for the localized generation of therapeutic agents, and is therefore of special interest for the release of anticancer drugs [3,4]. By uncaging the therapeutic molecule at the tumor site, off-target effects can be diminished [5]. Furthermore, the sustained generation of therapeutic molecules by bioorthogonal catalysts overcomes the payload limitations of traditional drug carrier strategies [6].

Transition metal complexes can catalyze bioorthogonal reactions [2], efficiently activating prodrugs. Non-native metals such as transition metals can, however, irreversibly bind to intracellular components and negatively impact the health of the cell [7]. The acute and long-term toxicity of many metals makes transition metal catalysts (TMCs) with low-toxicity metals important [8,9,10]. Gold(I)-based TMCs are a promising alternative to commonly used metal catalysts such as palladium-, gold(III)-, or ruthenium-based TMCs due to their low cyto- and genotoxicity [11,12,13,14,15]. Furthermore, gold(I)-based catalysts are highly specific toward alkyne activations and can therefore selectively activate therapeutic molecules or fluorophores [16].

Multiple water-soluble gold(I) TMCs have been developed for the bioorthogonal release of therapeutic agents [17,18]. However, many of these catalysts have low catalytic turnover number and frequency, which can be related to the instability of gold TMCs in aqueous solutions compared with organic solvents [16]. Furthermore, gold TMCs can experience rapid deactivation and degradation by endogenous molecules, such as thiols and proteins [19].

**Figure 1:**
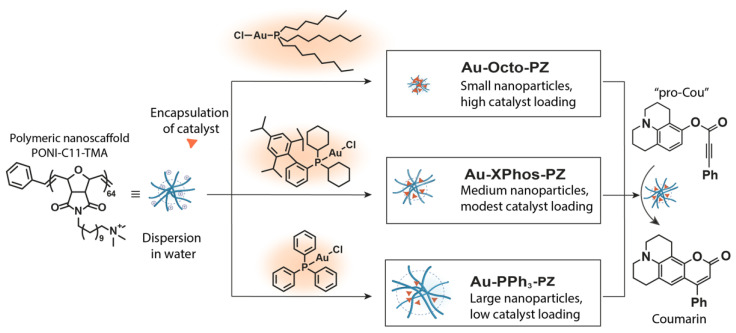
The encapsulation of gold TMCs into polymer scaffolds affords polyzymes. Variation in the ligand structure of the TMC leads to the generation of differently sized nanoparticles along with changes in the catalyst loading.

The embedding of TMCs into polymeric nanoscaffolds provides “polyzymes”, with the encapsulation protecting the catalyst from deactivation and degradation by endogenous molecules [6,20]. The polymeric scaffolds generally feature a hydrophobic interior and a hydrophilic exterior to provide water solubility [21]. We here report the co-engineering of a catalyst and polymer scaffold to achieve a high loading of the catalyst into the polyzyme with concomitant improvement in the catalytic activity. Gold(I)-phosphine catalysts were chosen as our model gold(I)-based TMC due to their ready synthesis and design versatility [22,23,24]. We tuned the hydrophobicity and molecular flexibility of the TMC by preparing three different polyzyme species based on gold(I)-complexes with different phosphine ligands. Our results indicate that the design of the TMC dramatically impacts the physical and catalytic properties of the polyzymes (Figure 1).

## 2. Materials and Methods

All chemicals and solvents used for the chemical synthesis were purchased from Fischer Scientific (Fisher Scientific International. Pittsburg, PA, USA) and Sigma-Aldrich (St. Louis, MO, USA). All solvents except for dichloromethane were used as purchased. Dichloromethane was freshly distilled from CaH_2_.

**Polymeric scaffold.** Polyoxanorborneneimide-C11-trimethylamine (PONI-C11-TMA) was selected as the polymeric nanoscaffold. A detailed procedure for the synthesis and characterization of this polymer via ROMP is provided in the (Supporting Information Figure S1a).

**Gold(I)-phosphine catalysts.** Chloro(triphenylphosphine)gold(I) (Au-PPh_3_) and chloro[dicyclohexyl [2′,4′,6′-tris(1-methylethyl)[1,1′-biphenyl]-2-yl]phosphine]gold(I) (Au-XPhos) were purchased from Sigma Aldrich. A detailed procedure for the synthesis and characterization of chloro(trioctylphosphine)gold(I) (Au-Octo) is provided in the Supporting Information.

**Pro-coumarin derivative.** A detailed procedure for the synthesis of 2,3,6,7-tetrahydro-1H,5H-benzo[ij]quinolizin-8-yl 3-phenyl-2-propynoate (pro-Cou) is provided in the Supporting Information.

**Catalyst encapsulation into the polymeric scaffold.** Each of the catalyst species was encapsulated into the PONI-C11-TMA scaffold using flash nanoprecipitation (FNP) [25,26]. For the encapsulation, 0.6 mg of the TMC (dissolved in 0.6 mL acetonitrile) was loaded into a 1 mL syringe. Into another 1 mL syringe, 0.6 mg of PONI-C11-TMA dissolved in 0.6 mL Milli-Q (MQ) water was loaded. Both syringes were attached to an FNP mixing device, and their contents were rapidly mixed by pushing the solutions into the FNP chamber (<1 s). The mixed solutions were ejected through an outlet of the chamber into a quench bath filled with 6 mL of MQ water. The loaded particles were transferred into a centrifuge filter (15 mL, Amicon Ultra, 10 kDa). The particles were washed five times by adding ~10 mL of MQ water, followed by concentration (2800 rpm, RT, 15 min). The resulting concentrated particles were filtered through a syringe filter (Millex-GP filter; 25 mm PES, pore size: 0.22 μm) to remove any unencapsulated precipitate of the catalyst, and the solution was then weighed. The mass concentration of the polyzymes was obtained by dividing the initially used mass of the polymer (0.6 mg) by the weight of the concentrated solution.

**Characterization of polyzymes.** The amount of encapsulated TMC within the polyzyme was determined via inductively coupled plasma mass spectrometry (ICP-MS), by detecting the amount of ^197^Au relative to the weight of the polymer according to previous protocols (Figure S2) [27]. The size and surface properties of the fabricated polyzymes were determined via DLS and measurement of the z-potential. The polydispersity index (PDI) was determined by independently measuring the size average of the particle three times and calculating the average of the PDI values. Furthermore, TEM images were obtained for all three polyzyme species. A detailed description of the measurement processes and z-potential results can be found in the (Supporting Information Figure S1b).

**Catalytic performance of the free catalyst molecules in organic solution.** Stock solutions of the free catalyst, pro-Cou, and AgOTf were prepared in CH_3_CN. Reagents were mixed in a vial to obtain final concentrations of 100 μM, 1 mM, and 80 μM respectively. AgOTf was used to aid with the abstraction of chloride from the gold center, which led to the activation of the gold catalyst [28,29]. The final solution was stirred at 37 °C for 3 h. The generation of fluorescence from the resulting coumarin derivative was measured (λ_ex_ = 402 nm, λ_em_ = 512 nm, cutoff = 495 nm) using a SpectraMax M2 microplate reader (Molecular Devices. San Jose, CA, USA). The catalytic yield was determined using a calibration curve of the fluorescent coumarin derivative in 100% CH_3_CN (Supporting Information, Figure S4).

**Kinetic performance of polyzymes in water.** Stock solutions of the polyzyme, glutamic acid, and AgOTf in MQ water were prepared to obtain final concentrations of 0.05 mg/mL, 0.1 mM, and 80 μM, respectively, in a 96-well black plate. Concentrations of the stock solutions were standardized according to the polymer concentration and therefore varied from the concentrations used during the free catalyst kinetics. The pro-Cou was dissolved in MQ water and CH_3_CN to yield a final composition of 98% MQ water and 2% CH_3_CN in the plate, due to its poor solubility in pure MQ water. The final concentration of pro-Cou in the plate was 10 μM. The kinetic behavior of the polyzyme toward the heterocyclization of the pro-Cou was measured by adding the pro-Cou, the respective polyzyme species, AgOTf, and glutamic acid to a single well. Negative controls contained (1) the polyzyme, glutamic acid, and AgOTf; and (2) pro-Cou, glutamic acid, and AgOTf. Each experiment was performed in triplicate at 37 °C. The generation of fluorescence was measured as described above. The catalytic yield was determined using a calibration curve of the fluorescent coumarin derivative in MQ water and 2% CH_3_CN, which can be found in the (Supporting Information Figure S3).

## 3. Results and Discussion

### 3.1. Physical Properties of Polyzymes

An amphiphilic polymer based on hydrophobic poly(oxanorbornene) with hydrophilic side groups was used to encapsulate the hydrophobic gold catalyst. In aqueous environments, this design enables the efficient stabilization of the TMCs into self-assembled nanoparticles [21]. The efficiency and utility of this system were demonstrated by Zhang et al. in previous studies [6,21]. The authors encapsulated a Pd-based TMC into poly(oxanorbornene)-based polymers, showing the activation of the drug mitoxantrone for the treatment of cancer cells [6]. These results inspired the search for novel TMCs based on nontoxic metals for the in situ activation of anticancer drugs. In particular, TMCs with high biocompatibility and catalytic turnover were desired. Gold(I)-based catalysts are particularly biocompatible and can specifically activate alkyne groups, which are functional groups not native to cells [16]. This particular reactivity can be used to perform hetero- and carbocyclization reactions, allowing for the assembly of the therapeutic molecules or fluorophores in situ.

The polymeric nanoscaffold used for the encapsulation of the herein-described TMCs features a hydrophobic oxanorborneneimide backbone and a C_11_ chain with a quaternary ammonium group, attached to the imide residue (Figure 1). In an aqueous environment, the hydrophobic parts of the polymer arrange to form a hydrophobic pocket. The terminal cationic headgroup at the end of the C_11_ chain provides water solubility [21].

Due to the hydrophobic nature of the polyzyme pocket, we hypothesized that a flexible structure of the organic ligand of the gold TMC would positively impact the properties of the polyzyme. Therefore, we encapsulated Au-PPh_3_, Au-XPhos, and Au-Octo (Figure 1) as the three gold TMCs with differences in structural flexibility. Au-PPh_3_ features freely rotatable phenyl groups, while Au-XPhos has a bulkier backbone. Au-Octo has freely rotatable alkyl chains.

**Figure 2:**
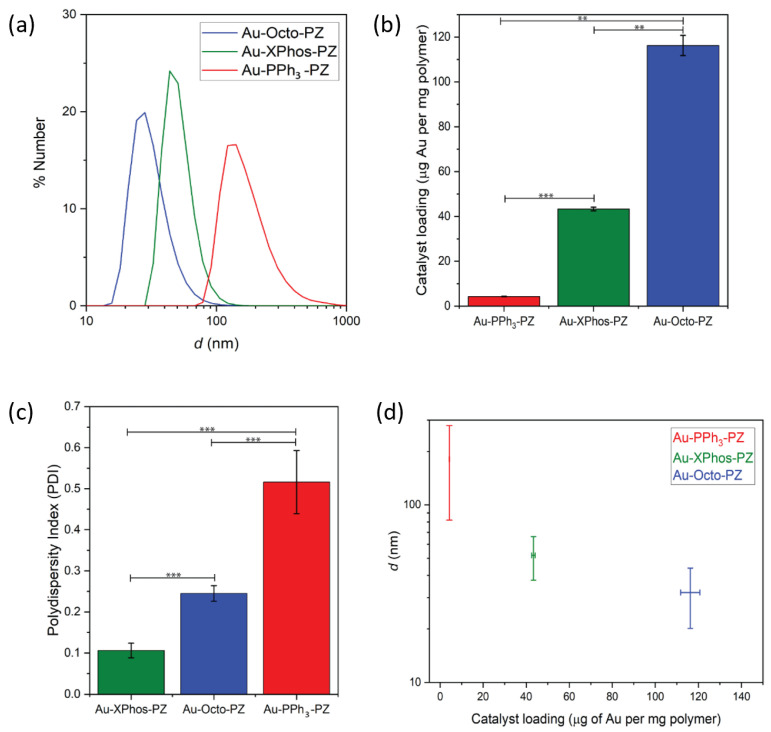
Characterization of the polyzymes. (**a**) Sizes of different polyzyme species were obtained via DLS measurements. (**b**) Catalyst loading was determined via ICP-MS, measuring the amount of gold present within the polyzymes and normalized to the mass of the polymer. (**c**) Polydispersity index (PDI) of polyzyme species. (**d**) Average polyzyme size versus catalyst loading. Measures were performed in triplicate, error bars represent the standard deviation. Statistical analysis was performed using Student’s *t*-test. ** = *p* < 0.05; *** = *p* < 0.005.

PONI-C11-TMA nanoparticles were characterized using DLS and zeta-potential measurements to compare the size of the polyzyme species with that of the empty polymeric nanoparticles. The average size of the dissolved nanoparticles was 1.6 ± 0.7 nm with a PDI of 0.7. We encapsulated the different TMC species into PONI-C11-TMA to formulate the polyzymes. A substantial change in the nanoparticle diameter between all three species was observed. Furthermore, a significant difference in the particle diameter compared with the empty nanoparticle scaffold was measured. The encapsulation of Au-PPh_3_ yielded particles of 180 ± 100 nm. The average size of the Au-XPhos-PZ was 52 ± 14 nm and that of the Au-Octo-PZ was 32 ± 12 nm (Figure 2a). Furthermore, the average polydispersity index (PDI) of the Au-PPh_3_-PZ was significantly higher than the PDI of the Au-XPhos-PZ and Au-Octo-PZ (Figure 2c). However, compared with the unloaded nanoparticle scaffold, a decrease in polydispersity was observed for all three polyzyme species.

The catalyst loading of each of the polyzyme species was then determined via ICP-MS, using ^197^Au, determining the ratio of encapsulated Au per weight of the polymer (Figure 2b). The results showed a large variation between the catalyst loading of the three different polyzyme species at the same polymer mass concentration. While Au-PPh_3_-PZ had the lowest catalyst loading, -XPhos-PZ had an ~8-fold higher catalyst loading, and Au-Octo-PZ had an ~23-fold higher catalyst loading compared with that of Au-PPh_3_-PZ.

**Figure 3:**
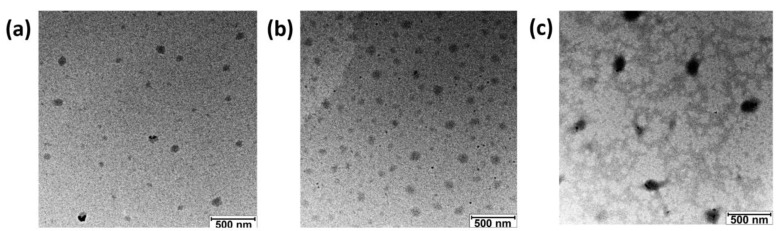
Characterization of polyzymes with encapsulated TMC species via TEM; (**a**) Au-Octo-PZ; (**b**) Au-XPhos-PZ; (**c**) Au-PPh_3_-PZ. Scale bar = 500 nm.

The characterization of the polyzymes using TEM confirmed the structural differences between each polyzyme species (Figure 3). While Au-Octo and Au-XPhos formed smaller and homogeneous spherical particles when encapsulated, Au-PPh_3_ formed larger and less homogeneous particles. These findings are consistent with results obtained via DLS.

The correlation of the average polyzyme size to the catalyst loading (Figure 2d) indicated that structural differences between the TMC species impacted the physical properties of the nanoparticles. While Au-PPh_3_ showed a relatively low encapsulation efficiency, both Au-XPhos and Au-Octo could be encapsulated in significantly higher amounts relative to the polymer weight. Furthermore, the polyzyme sizes strongly varied depending on the catalyst species.

### 3.2. Catalytic Performance of the Free TMCs and Polyzymes

We next compared the catalytic performance of the free TMCs in CH_3_CN with that of the encapsulated TMC species to determine how the encapsulation affected the catalytic behavior of the polyzymes. The catalytic performance of the free gold TMCs toward the heterocyclization reaction of pro-Cou (Figure 1) was quantified using fluorescence spectroscopy (λ_ex_ = 402 nm, λ_em_ = 512 nm). The catalytic yield of the free catalysts after three hours was measured (Table 1). The reaction was performed in CH_3_CN due to the insolubility of the TMC species in pure MQ water, using AgOTf as the chloride-abstraction agent. The catalytic performance of the gold catalysts in an organic solvent was expected to increase with a higher Lewis acidity of the gold center [30,31]. Triphenylphosphine and XPhos contribute significantly more to the Lewis acidity of the gold center than the trioctylphosphine ligand [32]. Consequently, Au-PPh_3_ and Au-XPhos produced a similar catalytic yield, while Au-Octo only produced a low yield of the coumarin derivative compared with the other TMC species.

**Figure 4:**
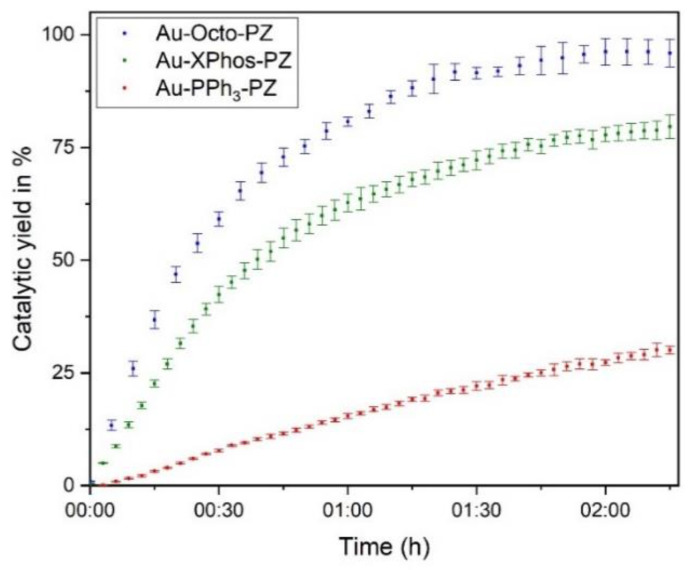
Kinetic behavior of the PZs toward the cyclization of the coumarin derivative, showing enhanced activity of Au-Octo-PZ polyzyme. The experiment was performed in triplicate. The error bars represent the standard deviation of all respective measurements.

We next encapsulated the TMCs into their respective polymer scaffolds as described, and performed the heterocyclization of pro-Cou (Figure 4). The experiment was performed in MQ water. Due to the insolubility of the pro-Cou in pure water, the substrate was first dissolved in CH_3_CN and subsequently added to the mixture, yielding a solvent composition of 98% MQ water and 2% CH_3_CN. Glutamic acid and AgOTf were added to abstract the chloride from the gold center and thereby activated the catalyst. Maintaining a low concentration of the polymeric vehicle is crucial because the toxicity of the nanocatalyst is primarily determined by the toxicity of the polymeric scaffold [33]. Therefore, the polyzyme species were consistently used at the same polymer concentration. The catalytic generation of the fluorophore was observed.

Due to the differences in the catalyst loading, the catalytic performance of the polyzyme species was expected to vary. A high catalyst loading led to higher catalytic turnover and yield. Furthermore, our observations indicated that the catalytic performance of the polyzyme did not correlate with the catalytic performance of the ‘naked’ TMC. While both Au-PPh_3_ and Au-XPhos reached a high catalytic yield in organic solution, Au-PPh_3_-PZ performed significantly worse when embedded into the polyzyme. However, Au-Octo-PZ performed significantly better than its free TMC counterpart. This behavior may be related to the encapsulation properties (Section 3.1). Due to the sensitivity of phosphine catalysts to oxygen and water, the catalytic activity of Au-Octo-PZ decreased over time, leading to the inactivation of the polyzyme after approximately 40 h (Figure S5). At the same time, no significant changes in particle size were observed (Figure S6), indicating the stability of the nanocomposite.

In summary, the simultaneous engineering of both the nanoscaffold and the encapsulated catalyst can lead to a collective improvement in bioorthogonal polyzymes. Our results highlighted the dependence of polyzyme efficiency on the catalyst structure. The modular scaffold provided by the polymer allows for the optimization of the catalyst loading by choice of the catalyst structure, reducing the quantity of polyzyme needed to perform efficient bioorthogonal catalysis. The high affinity between the flexible, aliphatic ligands of the metal complex and the hydrophobic structure of the polymer chain enhances the catalyst encapsulation and reactivity. As a result, the polyzyme prepared with the optimized catalyst showed improved TMC loading, stability, and catalytic activity, exceeding the efficacy of polyzymes prepared with commercial catalysts. Consequently, the modular assembly provided by the nanocomposite allows for easy optimization of the catalyst loading by choosing the most appropriate structure for the TMCs. The modularity and improved performance of these polyzymes amplify the potential for future cell-based and in vivo applications of bioorthogonal catalysts.

## Figures and Tables

**Table 1:** Maximum catalytic yield of free catalysts in organic solvent.

Catalyst Species	Au-PPh_3_	Au-XPhos	Au-Octo
Yield ^a^	73%	77%	29%

^a^ maximum yield achieved in CH_3_CN at 37 °C for 3 h with 80 μM AgOTf as an additive.

## Data Availability

Not applicable.

## Supplementary Materials

The following supporting information can be downloaded at: https://www.mdpi.com/article/10.3390/ma15186487/s1. Figure S1: (a) DLS size of PONI-C11-TMA; (b) z-potential of polyzymes (0.0015 mg/mL) in NaCl (5 mM) aqueous solution. Each measure was repeated three times, error bars represent the standard deviations. Figure S2: Calibration graph for ICP-MS measurement of ^197^Au. Figure S3: Calibration graph for coumarin derivative in 100% CH_3_CN. Figure S4: Calibration graph for coumarin derivative in MQ water and 2% CH_3_CN. Figure S5: Catalytic activity of AuOctoPhos polyzyme measured at different time points (red: 0 h, green: 20 h, blue: 40 h). The complete loss of catalytic activity of the is achieved after approximately 40 h. The experiment was performed in triplicate. The error bars represent the standard deviation of the measures. Figure S6: DLS size of AuOctoPhos polyzyme at different time points (dark blue: 0 h, blue: 20 h, cyan: 40 h). Figure S7: Enzyme kinetics of AuPPh_3_-PZ. The catalytic performance was determined using various substrate concentrations (10, 5, 2.5, and 1.25 μM, respectively). The PZ concentration and concentration of the additives (AgOTf and Glutamic acid) remained constant at 0.05 mg/mL, 80 μM, and 0.1 mM, respectively. Figure S8: Enzyme kinetics of AuXPhos-PZ. The catalytic performance was determined using various substrate concentrations (10, 5, 2.5, and 1.25 μM, respectively). The PZ concentration and concentration of the additives (AgOTf and Glutamic acid) remained constant at 0.05 mg/mL, 80 μM, and 0.1 mM, respectively. Figure S9: Enzyme kinetics of AuOctoPhos-PZ. The catalytic performance was determined using various substrate concentrations (10, 5, 2.5, and 1.25 μM, respectively). The PZ concentration and concentration of the additives (AgOTf and Glutamic acid) remained constant at 0.05 mg/mL, 80 μM, and 0.1 mM, respectively. References in Supplementary Materials can be found in [6,17,27].
