# Supplementary material for: Enhanced Design of Gold Catalysts for Bioorthogonal Polyzymes
Source: Materials (Basel). 2022 Sep 19;15(18):6487. doi: 10.3390/ma15186487 (PMC9506342; doi:10.3390/ma15186487)
Supplement: Supplementary file 1 [file materials-15-06487-s001.zip › materials-1805077-supplementary.pdf]

materials

Supplementary Material

Enhanced design of gold catalysts for bioorthogonal polyzymes

Cristina-Maria Hirschbiegel [1], Stefano Fedeli [1], Xianzhi Zhang [1], Rui Huang [1], Jungmi Park [1], Yisheng Xu [2] and Vincent M. Rotello [1,*]

[1] Department of Chemistry, University of Massachusetts Amherst, 710 North Pleasant Street, Amherst, MA 01003, USA
[2] State Key Laboratory of Chemical Engineering, East China University of Science and Technology, Shanghai, 200237 P. R. China
* Correspondence: rotello@umass.edu

1. Synthesis and characterization of compounds

^{1}H- and ^{13}C{^{1}H}-NMR spectra were obtained using a Bruker AVANCE 400 machine (400 MHz).

Polyoxanorborneneimide-C11-TMA.

The polymer scaffold was synthesized according to previous reports [1].

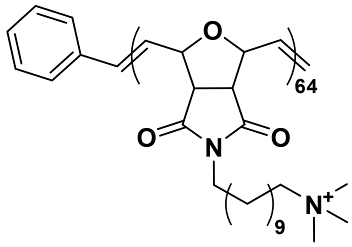

2,3,6,7-Tetrahydro-1H,5H-benzo[ij]quinolizin-8-yl 3-phenyl-2-propynoate (***pro-Cou***).

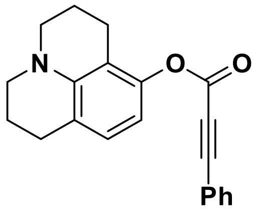

The compound was synthesized according to the literature procedure by Vidal et al. [2] ^{1}H NMR (400 MHz, CDCl3) δ 7.66 – 7.51 (m, 1H), 7.47 – 7.31 (m, 1H), 6.70 (t, J = 0.9 Hz, 1H), 6.12 (d, J = 8.1 Hz, 1H), 3.13 (q, J = 5.4 Hz, 5H), 2.77 – 2.71 (m, 3H), 2.68 (d, J = 6.7 Hz, 2H), 2.01 (dddd, J = 12.2, 11.2, 6.1, 3.3 Hz, 5H). ESI-MS [M+Na]$^{+}$ calc. 340.1, found: 340.2

Chloro(trioctylphosphine)gold(I) (***Au-Octo***)

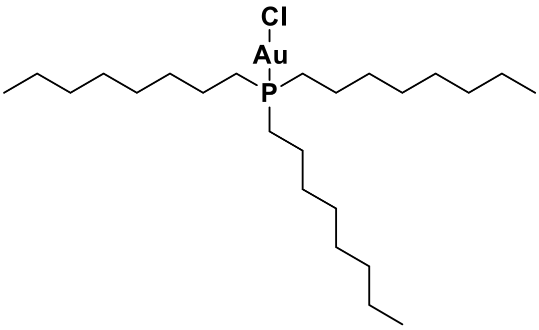

Tetrachloro auric acid (60 mg, 0.186 mmol, 1 eq.) was dissolved in ethanol and cooled to 0 °C. Trioctylphosphine (138 mg, 0.372 mmol, 2 eq.) was dissolved in ethanol and added dropwise to the solution under vigorous stirring. The solution was stirred at 0 °C until the yellow color disappeared. The ethanol was evaporated under reduced pressure. The obtained oil was redissolved in acetonitrile and the excess trioctylphosphine was extracted seven times with hexane. The acetonitrile was evaporated under reduced pressure, yielding a colorless oil (yield: 45 mg, 0.075 mmol, 40%). ^{1}H NMR ($CDCl_3$, 400 MHz): δ 0.88 (t, 9H), 1.32 (m, 30H), 2.03 (dd, 6H), 2.41 (p, 6H); $^{13}C\{^{1}H\}$ NMR ($CDCl_3$, 400 MHz): δ 13.96, 21.07, 22.49, 24.49, 25.55, 25.16, 28.88, 30.60, 30.74, 31.63. ESI-MS $[M-Cl]^+$ calc. 567.3, found: 567.4

2. Physical characterization of polymer and polyzymes

Hydrodynamic diameter and z-potential measurements.

Hydrodynamic diameter and z-potential of the polyzymes were measured by dynamic light scattering (DLS) in PBS using a Malvern Zetasizer Nano ZS instrument. The measurement angle was 173° (backscatter). Data were analyzed by the "multiple narrow modes" (high resolution) based on non-negative-least-squares (NNLS).

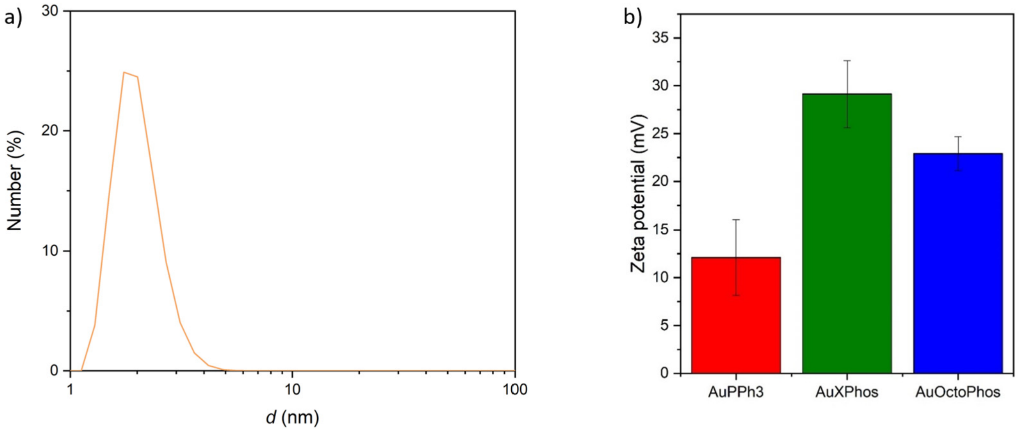

Figure S1. (a) DLS size of PONI-C11-TMA; **(b)** z-potential of polyzymes (0.0015 mg/mL) in NaCl (5 mM) aqueous solution. Each measure was repeated three times, error bars represent the standard deviations.

Transmission electron microscopy (TEM) measurements

TEM was carried out using a JEOL 2000FX operating at 200 keV for all three polyzyme species. Volumes of the turbid solutions were then dropcast onto a 300 mesh Cu TEM grid and dried for three days before the TEM analysis, respectively for all three PZ species.

ICP-MS characterization of loaded catalyst amount.

The catalysts embedded in the polyzymes were quantified, following previous protocols, [3] by diluting a sample of them in milli-Q water to a mass concentration of 0.05 mg/mL polymer. 20 µL of the diluted solution was taken for each replicate. 0.5 mL of aqua regia was added to each replicate and the samples were diluted to 10 mL by adding milli-Q water. The amount of encapsulated catalyst was measured using a Perkin-Elmer NexION 300X ICP mass Spectrometer, by tracking ^{197}Au and comparing it to the used polymer mass. For the calibration, a series of solutions with gold and (concentration: 0.2, 0.5, 1, 2, 5, 10, 20, and 50 ppb) were prepared (Figure 2). Operating conditions were as follows: nebulizer flow rate: 0.95 L/min; rf power: 1600 W; plasma Ar flow rate: 18 L/min; dwell time: 50 ms.

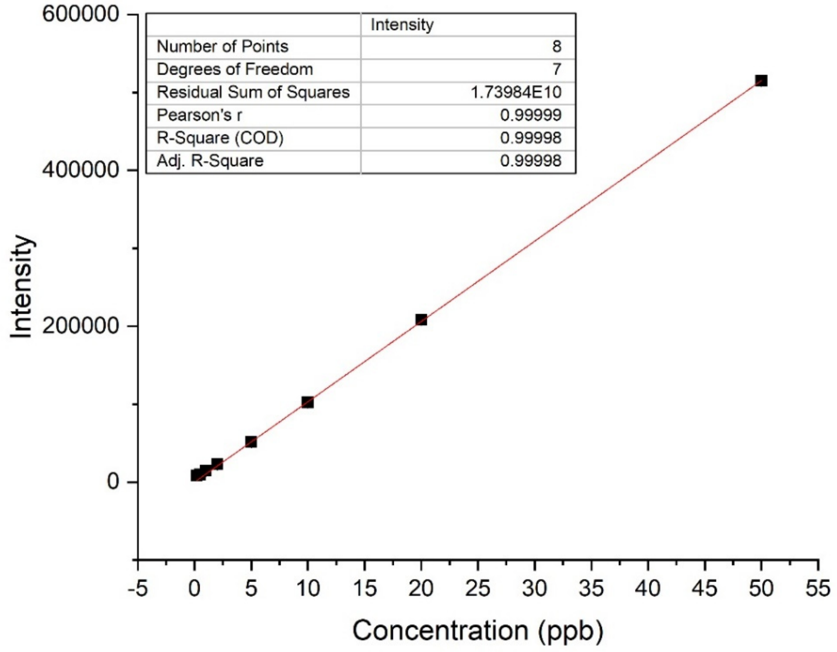

Figure S2. Calibration graph for ICP-MS measurement of ^{197}Au.

3. Fluorescence spectroscopy calibration graphs

For the determination of the yield of the fluorescent product, the following calibration curves were used.

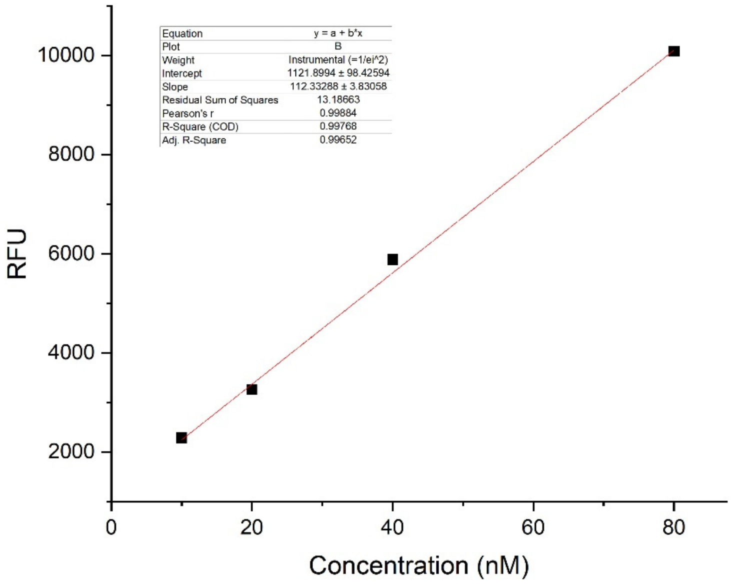

Figure S3. Calibration graph for coumarin derivative in 100% CH_3CN.

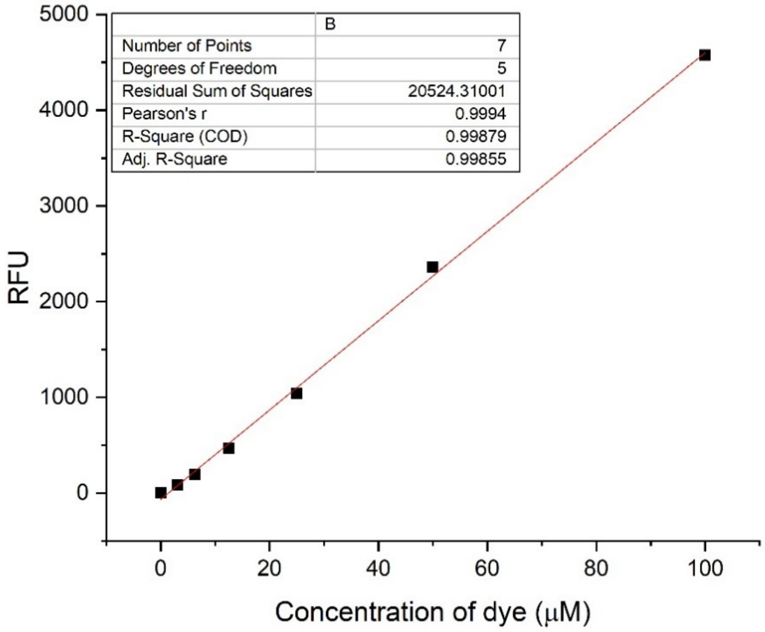

Figure S4. Calibration graph for coumarin derivative in MQ water and 2% CH_3CN.

4. Polyzyme stability

The catalytic activity of the highest active polyzyme (AuOctoPhos) was monitored at different time points (0 h, 20 h, 40 h). For all measurements, the same concentrations of pro-dye, AgOTf, glutamic acid, and polyzyme were used (10 μM, 1 mM, 80 μM, and 0.05 mg/mL, respectively). Furthermore, the size of the AuOctoPhos PZ was monitored via DLS at the same time points (0 h, 20 h, 40 h).

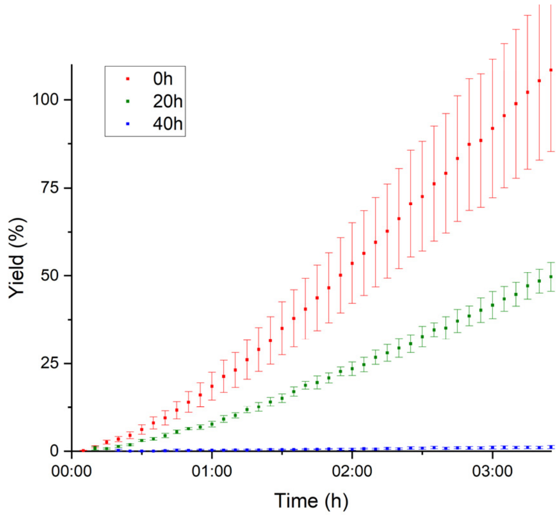

Figure S5. Catalytic activity of AuOctoPhos polyzyme measured at different time points (red: 0 h, green: 20 h, blue: 40 h). The complete loss of catalytic activity of the is achieved after approximately 40 h. The experiment was performed in triplicate. The error bars represent the standard deviation of the measures.

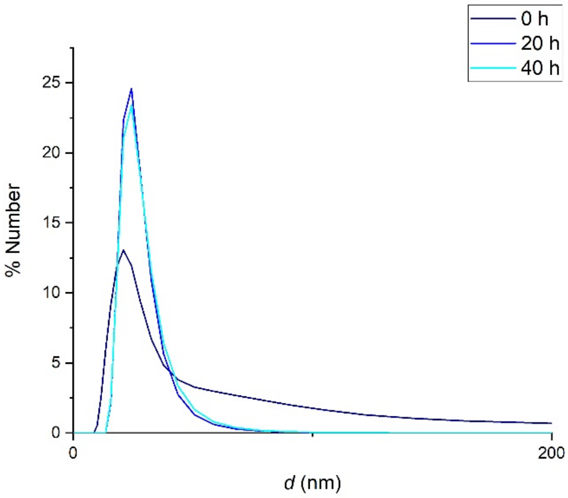

Figure S6. DLS size of AuOctoPhos polyzyme at different time points (dark blue: 0 h, blue: 20 h, cyan: 40 h).

5. Michaelis-Menten kinetics of PZs

The Michaelis-Menten kinetics of the different PZs were performed and v_{max} and K_m were determined. The respective Michaelis-Menten plots can be found in Figure S7 ($AuPPh_3$-PZ), Figure S8 (AuXPhos-PZ), and Figure S9 (AuOctoPhos-PZ).

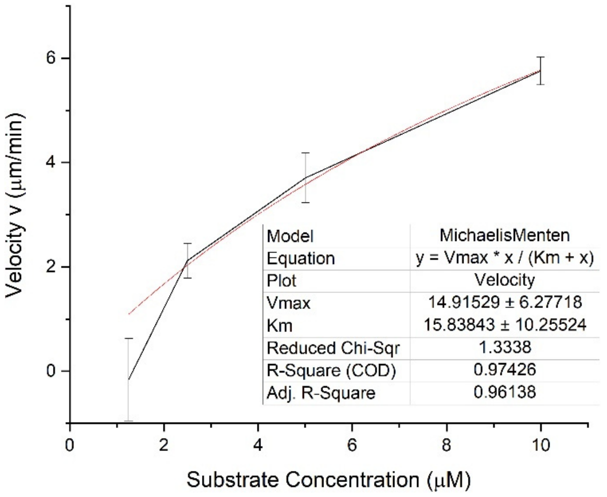

Figure S7. Enzyme kinetics of AuPPh3-PZ. The catalytic performance was determined using various substrate concentrations (10, 5, 2.5, and 1.25 µM, respectively). The PZ concentration and concentration of the additives (AgOTf and Glutamic acid) remained constant at 0.05 mg/mL, 80 µM, and 0.1 mM, respectively.

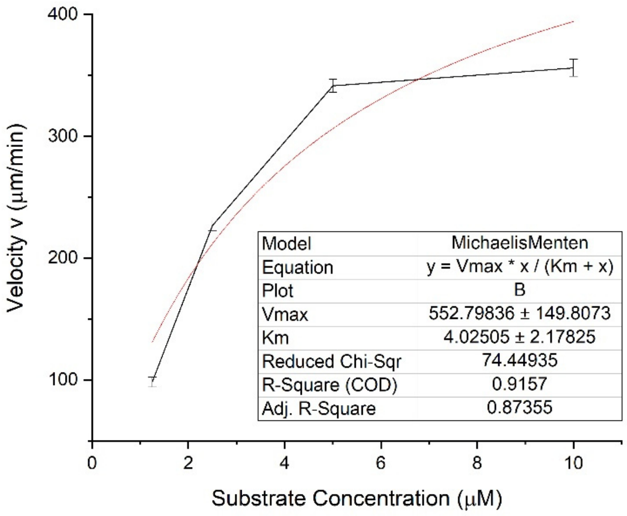

Figure S8. Enzyme kinetics of AuXPhos-PZ. The catalytic performance was determined using various substrate concentrations (10, 5, 2.5, and 1.25 µM, respectively). The PZ concentration and concentration of the additives (AgOTf and Glutamic acid) remained constant at 0.05 mg/mL, 80 µM, and 0.1 mM, respectively.

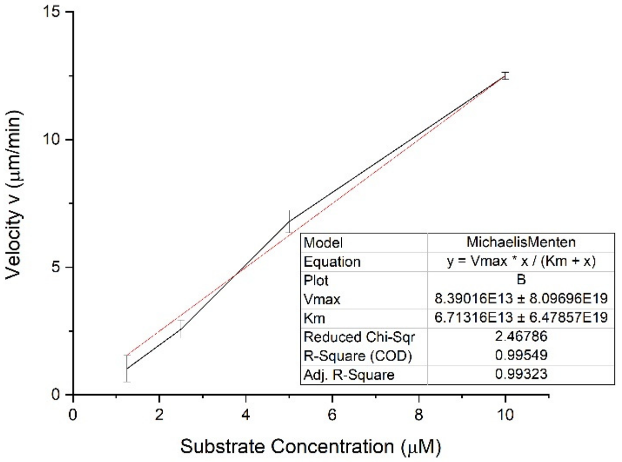

Figure S9. Enzyme kinetics of AuOctoPhos-PZ. The catalytic performance was determined using various substrate concentrations (10, 5, 2.5, and 1.25 μM, respectively). The PZ concentration and concentration of the additives (AgOTf and Glutamic acid) remained constant at 0.05 mg/mL, 80 μM, and 0.1 mM, respectively.

References

1 Zhang, X.; Landis, R. F.; Keshri, P.; Cao-Milán, R.; Luther, D. C.; Gopalakrishnan, S.; Liu, Y.; Huang, R.; Li, G.; Malassiné, M.; Uddin, I.; Rondon, B.; Rotello, V. M. Intracellular Activation of Anticancer Therapeutics Using Polymeric Bioorthogonal Nanocatalysts. *Adv. Healthc. Mater.* **2021**, *10* (5), 2001627.

2 Vidal, C.; Tomás-Gamasa, M.; Destito, P.; López, F.; Mascareñas, J. L. Concurrent and Orthogonal Gold(I) and Ruthenium(II) Catalysis inside Living Cells. *Nat. Commun.* **2018**, *9* (1), 1913.

3 Gupta, A.; Das, R.; Makabenta, J. M.; Gupta, A.; Zhang, X.; Jeon, T.; Huang, R.; Liu, Y.; Gopalakrishnan, S.; Milán, R.-C.; Rotello, V. M. Erythrocyte-Mediated Delivery of Bioorthogonal Nanozymes for Selective Targeting of Bacterial Infections. *Mater. Horiz.* **2021**, *8* (12), 3424–3431.
